# Structure of an octameric form of the minichromosome maintenance protein from the archaeon *Pyrococcus abyssi*

**DOI:** 10.1038/srep42019

**Published:** 2017-02-08

**Authors:** Giuseppe Cannone, Silvia Visentin, Adeline Palud, Ghislaine Henneke, Laura Spagnolo

**Affiliations:** 1 Institute of Molecular, Cell and Systems Biology, University of Glasgow, University Avenue, Glasgow G12 8QQ, UK; 2 School of Biological Sciences and Max Born Crescent, Edinburgh EH9 3JR, UK; 3 Centre for Science at extreme conditions, University of Edinburgh, Max Born Crescent, Edinburgh EH9 3JR, UK; 4 ISIS neutron source, Science and Technologies Research Council, Rutherford Appleton Laboratories, Harwell, OX11 0QX United Kingdom; 5 IFREMER, Laboratoire de Microbiologie des Environnements Extrêmes, UMR 6197, ZI de la pointe du diable CS 10070 29280 Plouzané, France; 6 Université de Bretagne Occidentale, Laboratoire de Microbiologie des Environnements Extrêmes, UMR6197, rue Dumont d’Urville 29280 Plouzané, France; 7 CNRS, Laboratoire de Microbiologie des Environnements Extrêmes, UMR6197, rue Dumont d’Urville 29280 Plouzané, France

## Abstract

Cell division is a complex process that requires precise duplication of genetic material. Duplication is concerted by replisomes. The Minichromosome Maintenance (MCM) replicative helicase is a crucial component of replisomes. Eukaryotic and archaeal MCM proteins are highly conserved. In fact, archaeal MCMs are powerful tools for elucidating essential features of MCM function. However, while eukaryotic MCM2-7 is a heterocomplex made of different polypeptide chains, the MCM complexes of many *Archaea* form homohexamers from a single gene product. Moreover, some archaeal MCMs are polymorphic, and both hexameric and heptameric architectures have been reported for the same polypeptide. Here, we present the structure of the archaeal MCM helicase from *Pyrococcus abyssi* in its single octameric ring assembly. To our knowledge, this is the first report of a full-length octameric MCM helicase.

Mini-chromosome maintenance (MCM) proteins are oligomeric enzymes that unwind the DNA double helix in an ATP-dependent manner. Homologues of eukaryotic MCM protein complex have been identified in all sequenced archaeal genomes, most of which have a single gene encoding for one MCM-like protein^1^. Among the exceptions, there are some Methanococcales, which possess between two and eight genes coding for MCM proteins^2^,^3^. In eukaryotes and archaea, MCM monomers are organized in three structural domains. The MCM amino-terminal domain (NTD) plays a role in higher order structure assembly and in the regulation of the ATPase activity, helicase activity and substrate specificity^4^. The AAA + module is needed to catalyse DNA unwinding^5^. Adjacent to the AAA + domain, the winged helix (WH) motif is proposed to have a regulative role. Many eukaryotic MCM2-7 subunits possess amino- or carboxy-terminal extensions involved in the regulation or recruitment of MCM2-7^4^. Important information on MCM helicases has been gathered from structural and functional studies of archaeal assemblies, as well as from the crystal structures of distantly related helicases (SV40, LTAg, and E1 helicase of bovine papilloma virus)^5^. A recent study of the human MCM helicase showed how this heterohexameric complex exhibits both ATP hydrolysis and DNA unwinding activities, and highlighted conformational changes on DNA binding^6^. A significant step forward in understanding the molecular organization and mechanism of the eukaryotic replicative helicase comes from the near-atomic resolution of the yeast MCM2-7 by cryo-electron microscopy. This model highlights the twisted and tilted single hexamers features of the MCM2-7 double rings and suggests a concerted mechanism for the melting of origin DNA that requires structural deformation of the intervening DNA^7^.

Archaea can provide a simplified model for understanding complex molecular machinery involved in DNA metabolism^8^. However, the differences between archaeal and eukaryotic replication machines should also be studied to provide additional information to our knowledge about this fundamental biological process, as well as to offer insights for possible biotechnological applications of unusual archaeal enzymes. The first structural observation of a multimeric assembly of MCM came from electron microscopy studies of *M. thermautotrophicus* MCM^9^. These studies showed a double hexameric structure with the two hexamers joined in a head-to-head manner, similar to the yeast MCM2-7 assembly observed later^10^. The human MCM helicase is a heterohexamer that exhibits intrinsic DNA unwinding activity^6^. MCM proteins were shown to assemble in several oligomeric states, including single hexamer, single heptamer, double heptamer and filaments^11^,^12^,^13^,^14^,^15^,^16^,^17^,^18^. Recently, a double octameric assembly has been reported for the ATPase domain of *Pyrococcus furiosus*^19^.

Here, we describe the structural analysis of a MCM-like protein, encoded by *Pyrococcus abyssi* (Pab) genomic ORF PAB2373. We cloned, overexpressed and purified *Pab*MCM, and we analysed it by single particle electron microscopy. We also tested its helicase activity *in vitro*. Our data show that *Pab*MCM is mainly arranged as a single octameric ring, with some degree of compositional heterogeneity, and its helicase activity is weaker compared to that of *Sso*MCM. This octameric assembly is different from the other full-length MCM helicases known to date, which are mainly hexameric and sometimes heptameric.

## Results

### Sequence analysis of the PabMCM’s AAA+catalytic domain

The sequence alignment of the archaeal MCM proteins from *S. solfataricus* (Q9UXG1), *M. thermoautrophicus* (O27798) and *P. furiosus* (Q8U3I4) with the predicted one from *P. abyssi* reveals high sequence homology (Figure S1) as well as showing the presence of two insertions that are predicted to be inteins. The first intein is inserted into the C-terminal of the Walker A motif, the second one is inserted at the N-terminus of the H2I-hp motif. *Sso*MCM and *Mth*MCM have no intein domains, while *Pfu*MCM has just one inserted before the H2I-hp motif. The N-terminal domain of *Pab*MCM reveals low sequence similarity with previously reported *S. solfataricus* and *M. Thermoautrophicus* MCM proteins (Figure S1). Biochemical and biophysical characterization demonstrated that the N-terminal domain plays a regulatory role in MCM function^20^,^21^,^22^ as well as being involved in the formation of single and the double ring assemblies^9^,^23^. The low sequence similarity of the N-terminus of the A and B domains could represent a form of adaptation to the extreme environment while the higher conservation of the C domain and the NCL linker a similar role in the ring formation and inter-subunits interaction, as previously seen for other MCM protein complexes. The AAA+ catalytic domain of *Pab*MCM is better conserved compared to the N-terminus (Figure S1). In *Pab*MCM, the active site (AAA+ domain) is ~250 residues long, and it is organized in Walker A, Walker B, sensor 1 (S1) and sensor 2 (S2) motifs. Sequence alignment shows that these motifs are well conserved in *Pab*MCM although polymorphisms are present in functional motifs such as EXT-hp, Walker A, H2I-hp, PS1-hp. These differences could represent mutations evolved as a form of adaptation to the extreme habitats in which *P. abyssi* thrives.

### Cloning of the intein-free PabMCM gene

**Figure 1:**
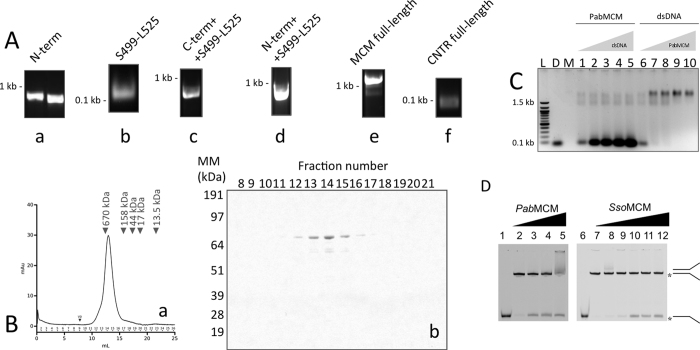
Cloning, purification and functional assay of PabMCM. (**A**) Reconstruction of the full-length PabMCM devoid of inteins. (a) PCR-amplified N and C-terminus. (b) PCR-amplified fragment Ser499-Leu525. (c) PCR-amplified of the joined N-term/Ser499-Leu525. (d) PCR-amplified of the joined N-term/Ser499-Leu525. (e) PCR-amplification of the *Pab*MCM full-length. (f) PCR performed in order to check that the fragment was successfully joined into the MCM sequence.(**B**) Size-exclusion chromatography. Gel-filtration was performed in 20 mM HEPES pH 7.4, 150 mM NaCl at 0.3 ml/min flow-rate. (a) Size-exclusion chromatography trace of PabMCM. The black arrow indicates the molecular weight of gel-filtration standards (BioRad). (b) SDS-PAGE analysis of eluted fractions. (**C**) DNA binding assay. The gel shows the PabMCM complex can bind dsDNA. A 59-mer dsDNA was used in this experiment. Size was chosen in order to avoid multi-MCMs loading on the same dsDNA and thus force a 1:1 stoichiometry. L, DNA ladder. D, dsDNA only used has control. M, PabMCM only used as control to avoid artefacts due to ethidium bromide staining. Lines 1–5 all contain 24 pmol (calculated on the monomeric MCM) of PabMCM with increasing amount of dsDNA (10, 20, 30, 40, 50 pmol). Lines 6–10 all contain 10 pmol of dsDNA with increasing amount of PabMCM (24, 48, 72, 96, 120 pmol). The band-shift experiment was performed as shown in Fletcher *et al*.^26^. (**D**) Helicase activity of PabMCM. Reactions contained helicase buffer with either no MCM (lanes 1 and 6, positive control or lanes 2 and 7, negative control) or increasing amounts of PabMCM (150, 300, 600 nM) lanes (3–5) and SsoMCM (50, 100, 150, 200, 300 nM) lanes (8–12).

The ORF PAB2373 (3336 bp) deposited at the NCBI data bank (http://www.ncbi.nlm.ni.gov) encodes a protein of 1112 amino acid residues, predicted to be a *Pab*MCM protein. The ORF contains two intein domains (Figure S1), whose insertion in the sequence cause the isolation of a fragment of the 27 amino acid residues. As result of this insertion, serine 499 is isolated from the adjacent lysine 525 of the predicted Walker A domain, while the predicted H2I-hp is not affected (Figure S1). According to the sequence alignment (Figure S1), the putative active full-length *Pab*MCM is devoid of inteins. In order to reconstruct the full-length gene, we used a PCR-based approach, in which fragments of the putative coding sequence were amplified separately and fused by PCR (Fig. 1A).

### Production, DNA binding and DNA Unwinding Activity of a Soluble PabMCM Complex

*Pab*MCM was purified by heat denaturation, followed by nickel affinity and size exclusion chromatographies (Fig. 1B). To ensure that no DNA contaminant was present in the sample, fractions eluted from the nickel affinity purification step were pooled and their A_260/280_ ratio measured spectrophotometerically. Only fractions with an A_260/280_ between 0.51 and 0.68 (99% protein) were concentrated and loaded onto a Superose 6 10/300 GL^TM^ size exclusion column. The apparent molecular weight of PabMCM, estimated as in ref. 24, was 575 kDa, indicating an octameric assembly.

Structural studies on archaeal MCM proteins have shown a positively charged central channel large enough to accommodate dsDNA^25^,^26^. In addition, biochemical studies reported that MCM proteins bind DNA through two types of structural motifs, which include a zinc finger and a beta-hairpin motif at the N-terminus and a beta-hairpin located in the AAA+ catalytic domain^22^,^27^,^28^. Bioinformatic analysis of the PabMCM sequence shows strong sequence homology and well-conserved structural motifs, with well-known archaeal MCM proteins (Figure S1).

Full-length *Pab*MCM binds dsDNA (Fig. 1C). The helicase activity of *Pab*MCM octamer complex has been examined by using a Y-shaped substrate. In the conditions tested *Pab*MCM had a mild (10% fork unwound) helicase activity, while our positive control *Sso*MCM exhibited maximal helicase activity (30% fork unwound) as previously shown^29^ (Fig. 1D). Similarly, *P. furious* MCM *(Pfu*MCM) shows weak helicase activity. However, it is stimulated upon interaction by GINS complex^29^. On the other hand, the unwinding activity of the thermophilic *M. thermoautotrophicum* MCM (*Mth*MCM) is stronger than the hyperthermophilic *Pfu*MCM and *Pab*MCM enzymes^8^. Moreover, it can unwind DNA substrates despite the presence of DNA-bound proteins^30^. Like *Mth*MCM, the thermophilic *Thermoplasma acidophilum* MCM (*Tac*MCM) displays significant helicase activity^31^. Studies carried out on the mesophilic *D. melanogaster* indicated that MCM2-7 has no activity in the absence of GINS and Cdc45^28^. Based on these comparative results, we conclude that *Pab*MCM is an active helicase.

### Electron microscopy Structure of Recombinant PabMCM

**Figure 2:**
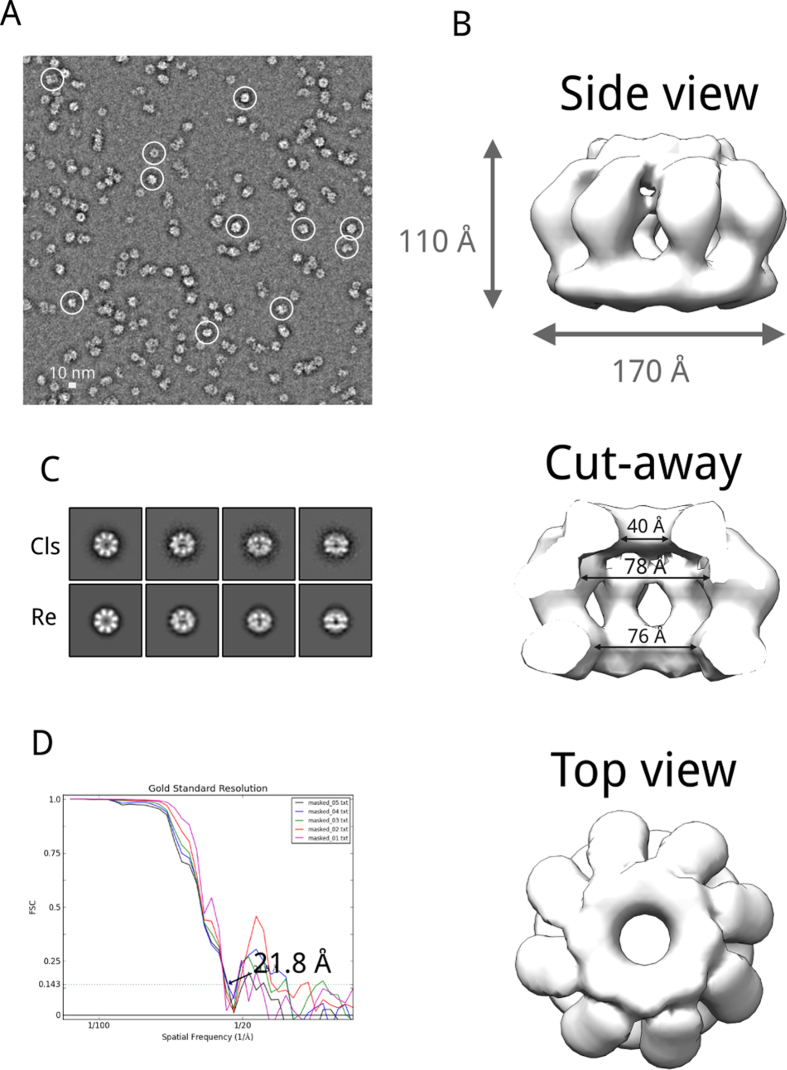
Electron microscopy and 3D refinement of the full–length *Pab*MCM. (**A**) Characteristic negatively stained electron micrograph of *Pab*MCM. Micrography recorded at 50,000x nominal magnification in low–dose mode (20–25 e^−^/Å^2^). White circles are used to show single particles. (**B**) Refined 3D model of the full-length *Pab*MCM single octamer. Volumes were rendered with Chimera^48^. (**C**) Class averages and re-projections for the refined 3D reconstruction. (**D**) Fourier shell correlation of the refined PabMCM model.

We used EM coupled to single particle analysis to glean insights into the three-dimensional architecture of the *Pab*MCM complex. We calculated a three-dimensional structure of the octameric assembly using single particles acquired in negative staining conditions. A typical micrograph is shown in Fig. 2A. ~45,000 molecular images (320 × 320 pixels, 1.51 Å/pixel) were pre-processed to a final size of 80 × 80 pixels at 6.04 Å/pixel. A first round of MSA classification was performed to calculate reference-free class averages and build a first catalogue of views present in the dataset. The resulting eigenimages, calculated by MSA, are shown in Figure S2. The eigenimages are normally presented in the order of their significance^32^. In this respect, eigenimage 2 reflects the characteristic of the main shape, which is a ring, in the dataset. Additionally, the strong black ring encircling the white ring is likely to be related to the size variation between particles into the dataset^32^,^33^. Eigenimages 3 and 4 have two-fold symmetry, which is likely to be related to a different oligomeric states (single/double ring)^33^. Eigenimages 5 and 6 reflect size variation for the tilted views. Eigenimage 7 shows a black and a white rings, probably still related to the different particles size within the dataset^32^. To check if this interpretation was correct, molecular images were classified using the first seven eigenimages. 100 class-averages were calculated (Figure S2). Analysis of these class-averages showed three types of class-averages: one of elongated shape and two ring-shaped (Figure S2B). The validity of the interpretation of the 2nd and 7th eigenimages was tested as in White, H.E. *et al*.^32^. Rotational averages of the circular and elongated class averages were calculated and then images were compared by subtraction. As shown in Fig. 2B, both eigenimages are representative of the size variation in the dataset. Based on this observation, the dataset was partitioned into two sub-datasets: the one containing elongated particles and the one with ring-shaped ones. Further rounds of MSA classification and alignment, with a subset of ~5,000 molecular images (10% of the total) classified as elongated, revealed their features. The elongated class averages show a two-fold symmetry and a four-tier organisation, which is the typical feature of a double ring assembly of *Mth*MCM, with the top and bottom tiers corresponding to the C-termini, and the two middle tiers corresponding to the N-termini^13^,^15^.

Analogous analyses were carried out for the 45,000-image subset of ring-shaped molecular images (90% of the total images). Further rounds of MSA classification led to classify two types of top-end views of *Pab*MCM: a small ring showing 7-fold symmetry and a bigger ring with 8-fold symmetry. The difference in size between the two rings was 8%. MSA classification also allowed sorting two types of side views, small and large side views. More difficult was the sorting of the tilted views since the difference in size was not always clear. From this dataset, ~10,000 molecular images belonging to the ‘large’ *Pab*MCM assembly were taken forward for calculating the initial 3D model of the full-length *Pab*MCM using EMAN2^34^,^35^ (Figures S3). The initial model was calculated with e2initial_model.py, which calculates a random blob model, from pure noise images, to seed a single particle reconstruction and refinement^35^. For this purpose, nine class averages were used for the 3D reconstruction of the initial model (Figure S3) and ten initial models were calculated. For each 3D reconstruction, the e2initial_model.py program calculates a set of re-projections, which can be used to estimate the quality of the 3D reconstruction. Each 3D reconstruction was checked by inspecting the 3D model and the correspondence between the class averages and the re-projections. The model with the best matching between class averages and re-projections was refined by projection matching with e2refine.py^35^. Re-projections and class-averages were checked at each iteration. Particular care was taken for the tilted views. The final refined model is shown in Fig. 2. Projections of the maps matched with 2D class-averages assigned the same Euler angles highlighting the validity of the map (Fig. 2C). The overall shape of the refined model *Pab*MCM exhibits similarity with 3D-EM models previously reported archaeal MCMs. However, the *Pab*MCM complex exhibits an octameric assembly and has overall dimensions of 170 Å × 170 Å × 110 Å. The resolution of the map is 22 Å, calculated at 0.143 Fourier Shell Correlation (FSC) (Fig. 2D).

To test that the conditions used for negative staining did not affect the assembly of *Pab*MCM, we carried out preliminary cryo-EM experiments (Figure S4, panels A and B). We confirmed that single *Pab*MCM octamers also exist in cryo conditions. ~33,000 particles (Figure S4C) were manually picked and used to feed a 2D classification experiment in a semi-automated manner, using the Relion software^36^ without imposing symmetry at any stage. The classes calculated for the whole dataset clearly show the coexistence of single ring as well as higher-order assemblies (Figure S2E). Among the single ring assemblies, we identified a majority of rings bearing 8-fold symmetry. Particles belonging to these classes were extracted from the full dataset and used as an input for reference-free 2D classification (Figure S4). These classes have features that are in excellent agreement with the ones obtained in the negative staining structure, showing that the octameric assembly is not an artifact of negative staining experiments (Fig. 2C).

### Interpretation of the PabMCM 3D-EM structure

**Figure 3:**
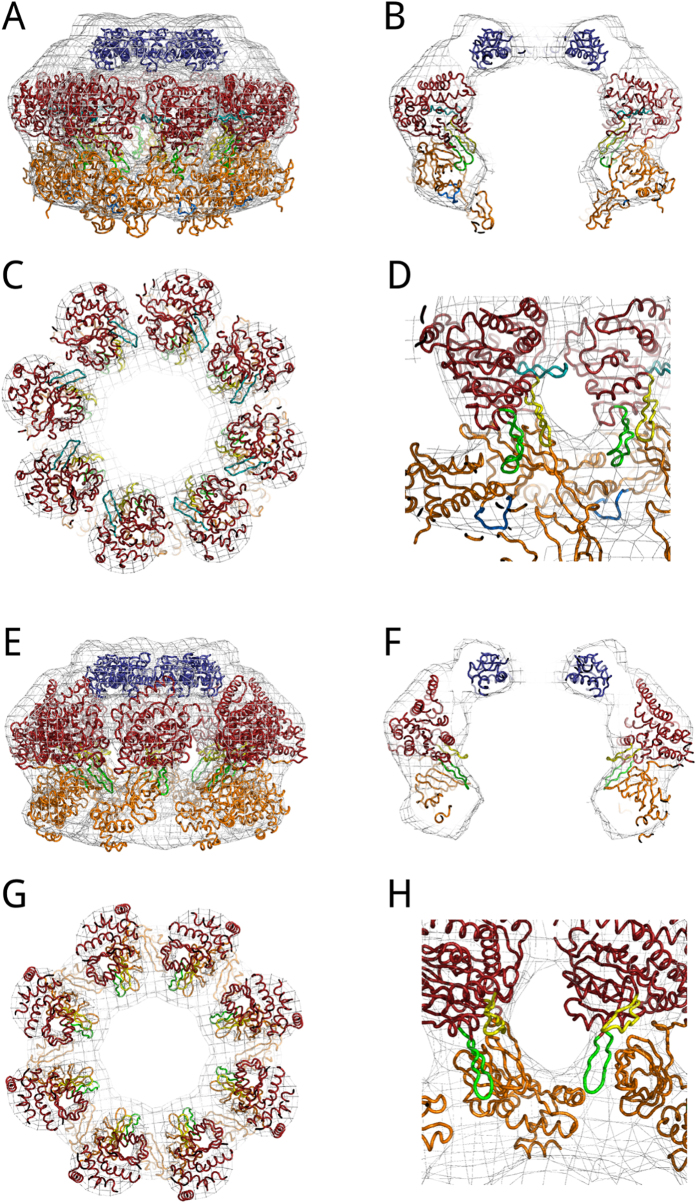
Model fitting of the full–length PabMCM 3D EM structure. (**A,B,C,D**) Fitting of the atomic coordinates of the C–terminally truncated *Sso*MCM (PDB 3F9V)^25^ and C–terminal domain of *Sso*MCM (PDB 2M45)^37^. (**E,F,G,H**) Fitting of the atomic coordinates of full-length *Mka*MCM (PDB 3F8T)^16^. In blue is shown the fitting of the NMR structure of *Sso*MCM. In red, the AAA + module while in orange the N-terminal domain of both *Sso*MCM and *Mka*MCM. In light blue, EXT β-hairpin; in yellow, H2I β-hairpin; in green, PS1 β–hairpin; in dark blue, NT β–hairpin (only in panel D).

To interpret the 3D-EM reconstruction of the full-length *Pab*MCM, we docked the crystal structure of the C-terminally truncated *Sso*MCM^25^; the crystal structure of the full-length *Mka*MCM^16^ and the NMR structure of the C-terminal domain of *Sso*MCM^37^. The fitting of the 3D structure of the full-length *Pab*MCM complex (Fig. 3) was performed manually using Chimera^38^ and then optimised with Situs^39^. 3D volume and fitted model were rendered in Pymol^40^. The crystallographic structure of the C-terminally truncated SsoMCM fitted well into the electron density corresponding to the bottom and top tier, although a C-terminal extra density was observed (Fig. 3A and B). The orientation resulting from docking of *Sso*MCM into the map shows PS1 and HP2 β-hairpins pointing into the central channel, while Ext β–hairpin is pointing toward the side channel between the subunits composing the ring (Fig. 3C and D). Ext β-hairpin locates on the exterior side of the side channel (close-up Fig. 3D). The crystallographic structure of the full-length *Mka*MCM monomer fitted better into the electron density corresponding to the bottom and top tier (Fig. 3E and F). Docking resulted in PS1 and HP2 β–hairpins of *Mka*MCM pointing into the central channel of the 3D-EM map of the full-length *Pab*MCM (Fig. 3G and H).

**Figure 4:**
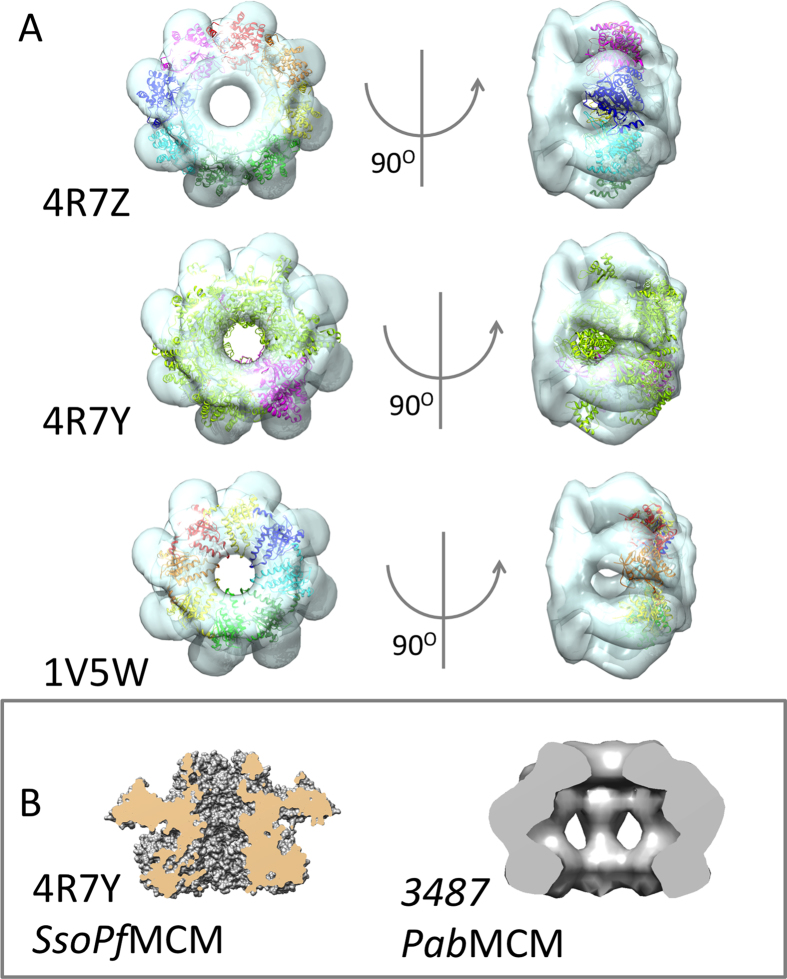
Structural superposition. (**A**) One octameric PfuMCM AAA ring (PDB 4R7Z)^19^, the N-terminal tier of the hexameric *PfuSso*MCM chimera (PDB 4R7Y)^19^ and the human Dmc1 recombinase (PDB 1V5W)^41^ superposed on PabMCM highlights the similarity with the symmetry and AAA ring subunits orientation of the crystallographic structure for PfuMCM. (**B**) Comparison of the central channel of the hexameric *Sso*MCM (47RY) and the octameric *Pab*MCM (EMD-3487).

The structural superposition of one octameric *Pfu*MCM AAA ring (PDB 4R7Z)^19^ on the middle layer of the octameric *Pab*MCM shows that the diameter of the central cavities is very similar in both structures (Fig. 4A). Despite their different oligomeric state, the diameter of the N-terminal tier of the hexameric *PfuSso*MCM chimera (PDB 4R7Y)^19^ also almost fits the top density, however it is slightly wider. On the other hand, the comparison with another octameric AAA assembly involved in DNA metabolism, the human Dmc1 recombinase (PDB 1V5W)^41^ highlights that in this case the upper tier of the *Pab*MCM channel is slightly wider than that of the recombinase (Fig. 4A). The best fit for symmetry and cavity dimension could therefore be represented by a possible octameric ring of eight full-length *Mka*MCM subunits, that crystallised as monomers in 3F8T^16^. The milder activity of PabMCM compared to that of SsoMCM could be related to structural differences such as the broader end of the cavity, possibly due do the octameric arrangement of the subunits in the ring. It should however be noted that the length of each individual subunit in the octamer is not more extended than that of each of the eight modeled full-length subunits of MkaMCM (PDB 3F8T)^16^. The comparison of the hexameric *Sso*MCM and the octameric *Pab*MCM channels (Fig. 4B) shows two very different overall shapes. This different arrangement could be one explanation for the different activities of the two helicases.

## Conclusions

The structural observation of archaeal MCM proteins by electron microscopy showed that several oligomeric states could be adopted by MCM proteins, including single hexamer, single heptamer, double heptamer and filaments^9^,^10^,^11^,^12^,^13^,^14^,^15^,^16^,^17^,^18^,^19^.

In this study, we report the structure of a homo-octameric MCM protein. Electron microscopy coupled to SPA revealed that *Pab*MCM is a mixture of at least three molecular species: single heptamer, single octamer and double rings. A subset of ~10,000 particles, showing characteristic 8-fold symmetry, were classified and sorted by MSA. This subset was homogeneous enough for a subsequent 3D reconstruction. The final 3D-EM map at 22 Å (calculated by FSC) showed similarity with previously reported 3D-EM reconstruction of similar archaeal MCM proteins, however this is the first non-chimeric MCM helicase reported to assemble as an octamer.

*Pab*MCM binds to dsDNA (Fig. 1C), and has weak helicase activity compared to *Sso*MCM (Fig. 1D). Studies of another hyperthermophilic archaeal MCM, *Pfu*MCM, demonstrated that the helicase activity of MCM is stimulated by the GINS complex^42^. Like other hyperthermophilic MCM such as *Thermococcus Kodakaraensis* MCM1^2^ or *Pfu*MCM, the helicase activity seems to be reduced. Additional replication factors like the GINS complex and cdc6^42^,^43^ or post-translational modifications^44^ are described to further enhance the helicase activity. Hence, similar mechanisms of activation could be involved in *Pab*MCM functionality. The structure for the full-length PfuMCM assembly is not yet available, however it is noteworthy that the octameric arrangement of PabMCM is very reminiscent of that of the PfuMCM AAA ring, suggesting an interesting similarity between two Pyrococcal proteins. The weak helicase activity could be due to the conformation of the bell-shaped inner cavity (Fig. 4B) which is broader than that of hexameric helicases such as SsoMCM (Fig. 4B).

## Experimental Section

### Cloning

The cloning was performed from Pab genomic DNA (kind gift of P. Forterre). The primers oligo1 and oligo4 were used to PCR-amplify the N-terminus of MCM, the primers oligo5 and oligo6 were used to amplify the fragment Ser499-Leu525 and the primers oligo3 and oligo2 were used to amplify the C-terminal fragment of MCM (Supplementary Table 1). The full-length *Pab*MCM was reconstructed in three PCR reactions (Fig. 1). In each reaction, the PCR to join the fragments was performed in two steps. In the first step, two fragments were incubated in the reaction mixture for 5 cycles, in absence of primers, in order to fill the 5′ ends. In the second step, primers were added and PCR was performed for 35 cycles. In the first PCR, the N-terminal fragment was fused with the fragment Ser499-Leu525 and then amplified by the Oligo1 and Oligo6 pair. In the second PCR, the fragment Ser499-Leu525 was fused with the C-terminal fragment of MCM and then amplified by the pair of oligos 5 and 2. The reconstructed full-length MCM fragment was cloned into a modified pET vector and the sequence was confirmed by sequencing.

### Protein overexpression and purification

*Pab*MCM was overexpressed in Rosetta (DE3) pLysS grown in terrific broth (TB). Cells were grown to Abs_600_ = 0.4–0.6 before induction with 0.2 mM IPTG for 16 hours at 30 °C. *Pab*MCM was purified by heat–denaturation (20′ at 70 °C), followed by nickel affinity chromatography and size-exclusion chromatography. Fractions eluted from the nickel affinity purification step were pooled and tested for DNA contaminations by measuring the Abs_260_/_280_ ratio. Only fractions with Abs_260_/_280_ ratio between 0.51 and 0.68 (99% protein) were concentrated by ultra-filtration using a 15 R Vivaspin (30,000 MWCO), to a final concentration of 15 mg/ml. Finally, the sample was loaded onto a Superose6^TM^10/300 GL size-exclusion chromatography column. The apparent molecular weight of PabMCM, estimated as in Andrews^24^, was ~575 kDa.

### DNA binding assay

DNA binding assays were carried out as described in ref. 25. Oligos 3 and 4 were annealed in annealing buffer (50 mM Tris, 50 mM NaCl, pH 8.8) to a final concentration of 10 μM. Annealing was achieved by incubating the tube containing the oligo mixture in a beaker with boiling water and letting it cool overnight. Reactions were carried out at 50 °C for 30 minutes. Annealing was checked by electrophoresis onto 1.5% agarose gels.

### Helicase assay

The fluorescent-labeled Y-shaped substrate is obtained by mixing oligos A and B (Supplementary Table 2) as described in DNA binding assay and followed by PAGE purification.

The helicase assays (20 μl) were performed by mixing 5 nM fluorescent-labeled Y-shaped substrate, 10 mM ATP, and increasing amounts of enzyme in reaction buffer containing 20 mM Tris-acetate pH 7.9, 50 mM potassium-acetate, 10 mM magnesium-acetate, and 1 mM DTT. Reactions were initiated by the addition of ATP, and the mixtures were incubated at 65 °C for 45 minutes. Reactions were terminated by the addition of 11 μl of chilled stop solution (0.5 mg/ml Protéinase K, 0.5% [wt/vol] SDS, 5 mM EDTA, and 250-fold molar excess of unlabeled DNA trap oligonucleotide C to minimize reannealing of the unwound oligonucleotides. Followed by incubation at room temperature for 15 minutes, 4 μl loading buffer are added to the samples. As a positive control, DNA substrates were heat denatured at 95 °C for 15 min in the absence of helicase. As a negative control, the substrates were incubated in the reaction mix in the absence of helicase. Samples were resolved in a 8% (vol/vol) polyacrylamide gel in 1 × Tris-borate-EDTA. The fluorescent-labeled bands were visualized using a Typhoon 9400 (GE healthcare) and quantified with ImageQuant 5.2 software (Molecular Dynamics).

### Electron Microscopy

*Pab*MCM was studied both by negative staining and cryo electron microscopy (EM) and single particle analysis (SPA). The protein sample was added to glow-discharged grids, blotted and washed twice before staining using 1% uranyl acetate. Data were collected on a FEI F20 FEG microscope, equipped with a TemCam-F816 (8kx8k) CCD camera. Images were collected under low dose mode at nominal magnification of 50,000x, at a final sampling of 1.51 Å/pixel at the specimen level. Negatively stained single particle images were selected interactively using the e2boxer.py program from the EMAN2 single particle analysis package and extracted into boxes. Image processing was performed using the IMAGIC-5^45^ and Eman^35^,^46^ packages. The dataset was re-sampled at 6.04 Å/pixel. ~45,000 images with homogeneous staining were band-pass filtered with a high pass cutoff of 110 Å and a low pass cutoff of 18 Å. The single particle images were analysed by Multivariate Statistical Analysis with IMAGIC-5. The dataset was subjected to successive rounds of alignment and classification in order to improve the resulting image class-averages. Three-dimensional reconstruction was performed using Imagic and Eman2 protocols. The map was deposited in the PDB with accession code EMD-3487. The cryoEM data collection was performed on Quantifoil grids covered with a thin layer of carbon, plunge-frozen into liquid ethane using a Vitrobot instrument. Data collection was performed at the same magnification of the negative staining study (50,000x), in low-dose mode. The single particle analysis was performed using the Relion software^36^ according to instructions. Figures were prepared with UCSF Chimera^38^ and Pymol^47^.

## Additional Information

**How to cite this article**: Cannone, G. *et al*. Structure of an octameric form of the minichromosome maintenance protein from the archaeon *Pyrococcus abyssi. Sci. Rep.* **7**, 42019; doi: 10.1038/srep42019 (2017).

**Publisher's note:** Springer Nature remains neutral with regard to jurisdictional claims in published maps and institutional affiliations.

## Supplementary Material

Supplementary Figures
