# Supplementary material for: Structure of an octameric form of the minichromosome maintenance protein from the archaeon Pyrococcus abyssi
Source: Sci Rep. 2017 Feb 8;7:42019. doi: 10.1038/srep42019 (PMC5296750; doi:10.1038/srep42019)
Supplement: Supplementary Figures [file srep42019-s1.pdf]

Supplementary material:

Structure of an octameric form of the minichromosome maintenance protein from the archaeon *Pyrococcus abyssi*

Giuseppe Cannone[1,2,3] Silvia Visentin[1,2,4], Adeline Palud[5,6,7], Ghislaine Henneke [5,6,7] and Laura Spagnolo[1]*

[1]Institute of Molecular, Cell and Systems Biology, University of Glasgow, University Avenue, Glasgow G12 8QQ; [2]School of Biological Sciences and [3]Centre for Science at extreme conditions, University of Edinburgh, Max Born Crescent, Edinburgh EH9 3JR, UK; [4]ISIS neutron source, Science and Technologies Research Council, Rutherford Appleton Laboratories, Harwell, OX11 0QX United Kingdom [5]IFREMER, Laboratoire de Microbiologie des Environnements Extrêmes, UMR 6197, ZI de la pointe du diable CS 10070 29280 Plouzané, France; [6]Université de Bretagne Occidentale, Laboratoire de Microbiologie des Environnements Extrêmes, UMR6197, rue Dumont d'Urville 29280 Plouzané, France ; [7]CNRS, Laboratoire de Microbiologie des Environnements Extrêmes, UMR6197, rue Dumont d'Urville 29280 Plouzané, France

*Corresponding author: laura.spagnolo@glasgow.ac.uk

Cannone *et al.*, Figure S1

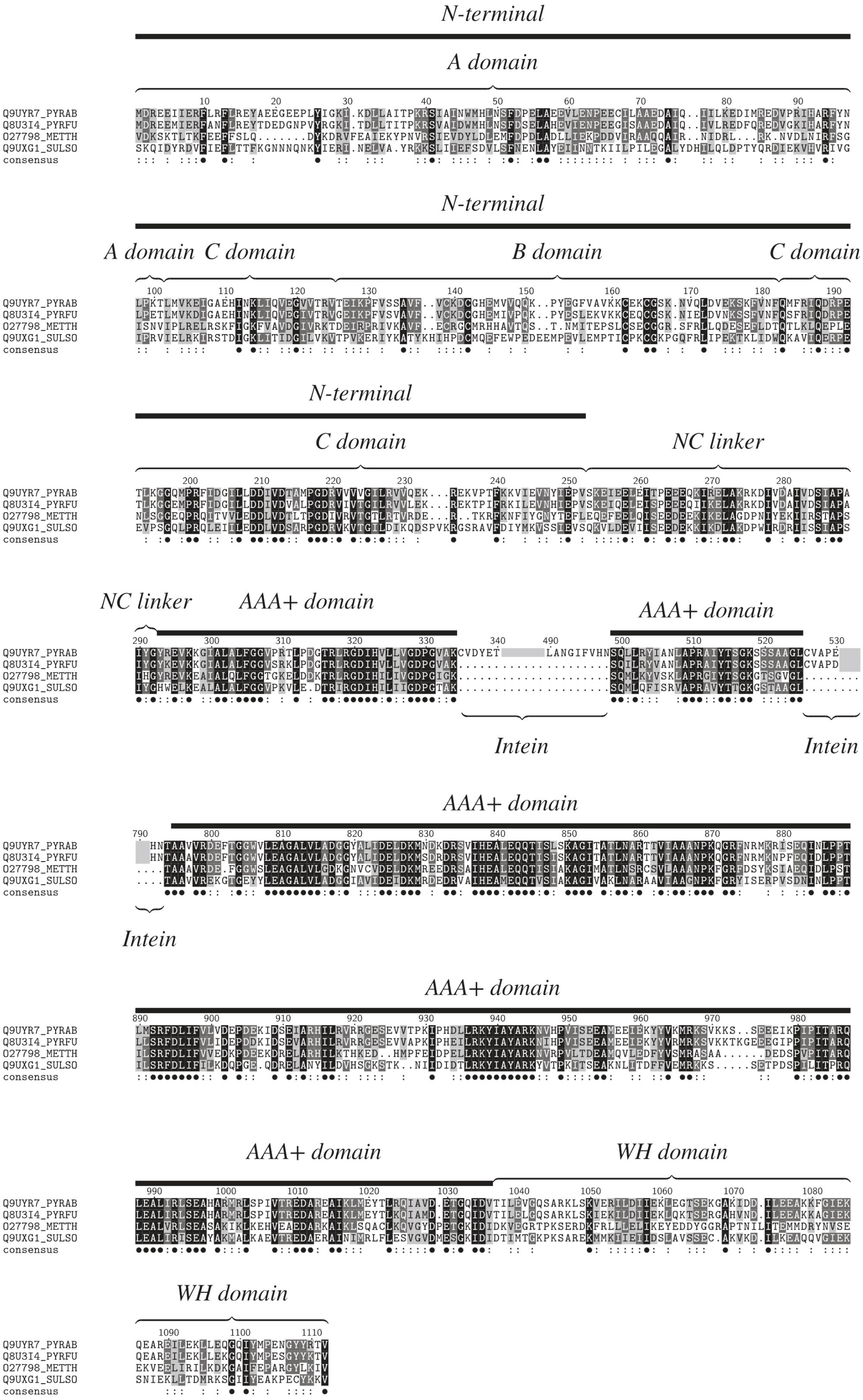

Cannone *et al.*, Figure S2

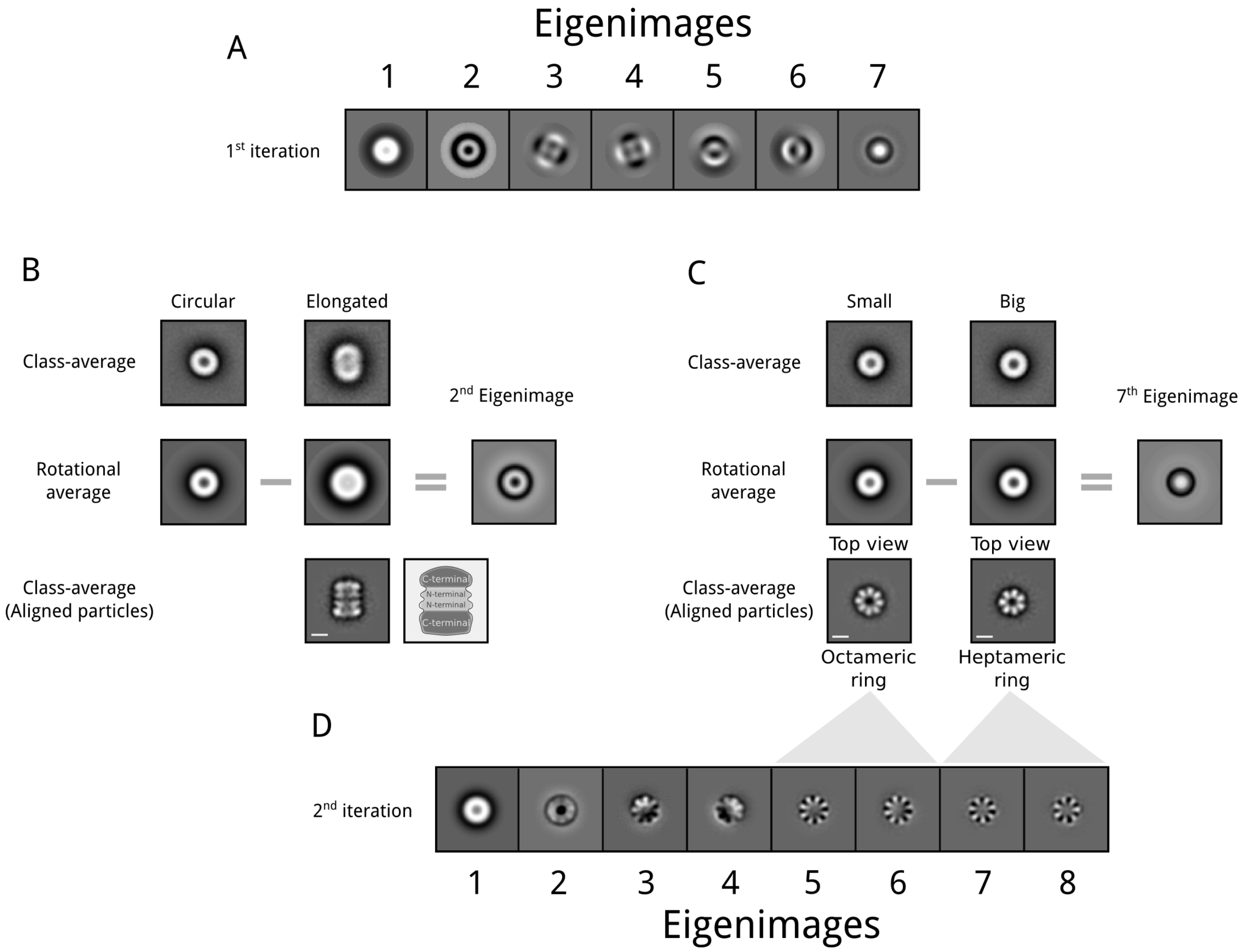

Cannone *et al.*, Figure S3

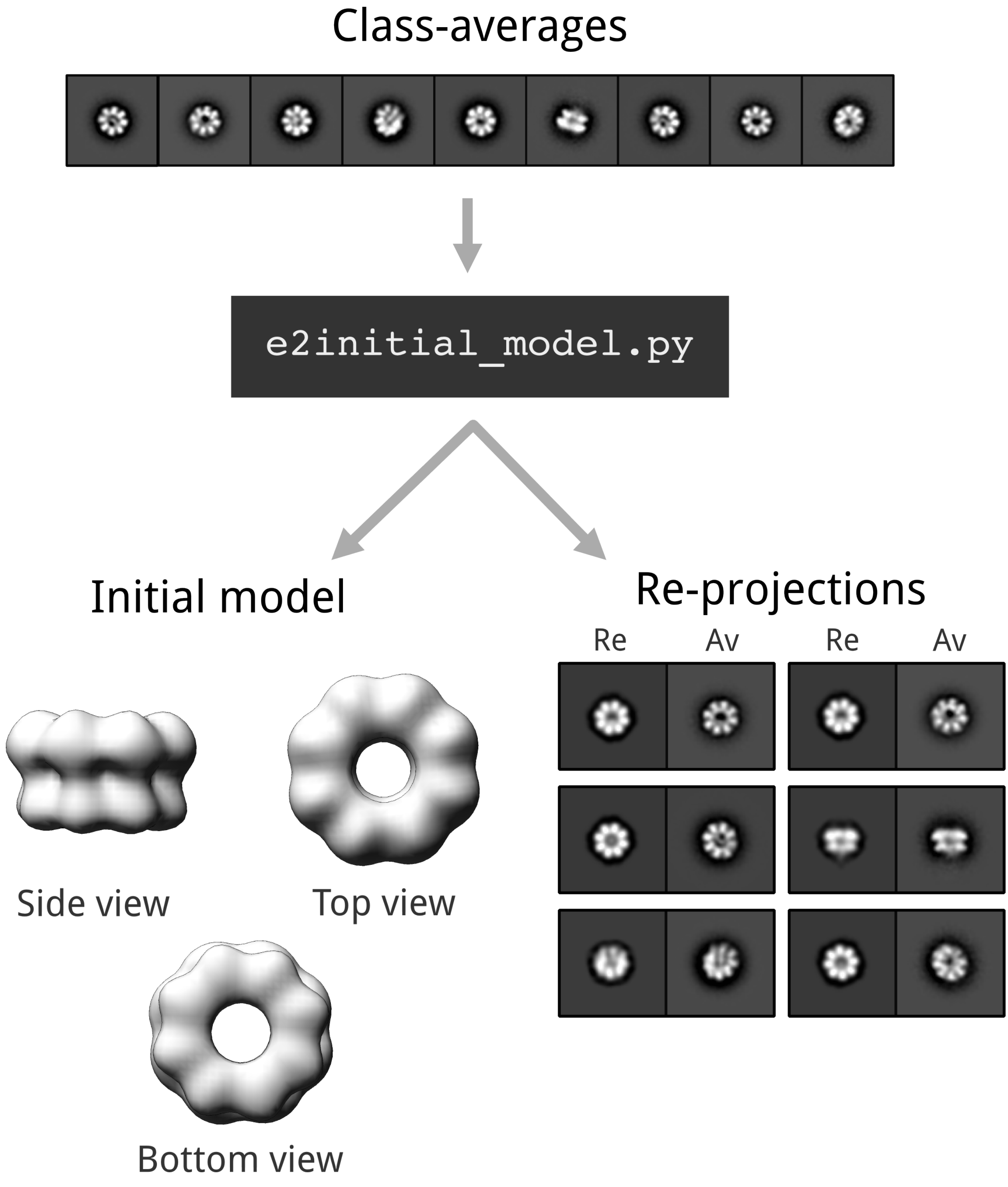

Cannone *et al.*, Figure S4

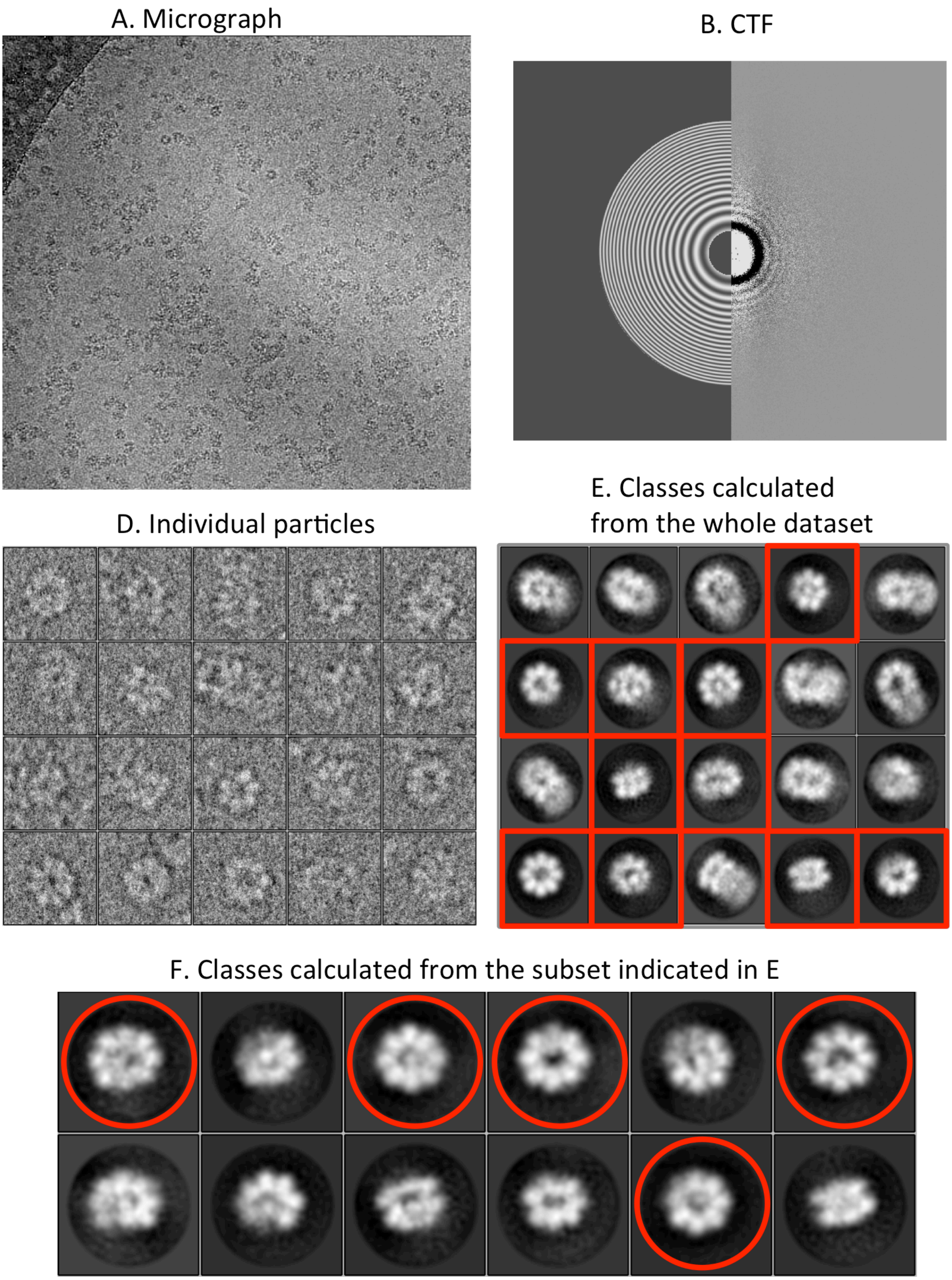

Supplementary Figures Legends

Figure S1: Sequence alignment of *S. solfataricus*(Q9UXG1), *M. Thermoautrophicus* (O27798), *P. furiosus*(Q8U3I4) and *P. abyssi* MCMs. Highly conserved residues (indentity 100%) are shaded in black while black dots are shown in the consensus. Residues with indentity >75% are shaded in light grey.

Figure S2: MSA classification of *Pab*MCM molecular images. (A) First eigen images used for the classification of the molecular images. (B) Two characteristics class-average, which show size variation. Class averages were compared by subtraction (Circular minus elongated) to verify that the eigenimage 2 was representative of the difference in size between circular and elongated particles. Further MSA classification followed by multi–reference alignment (MRA) of the molecular images belonging to the elongated class–average revealed a characteristic two–fold symmetry and four tiers structure. These features are similar to those ones previously observed for *Mth*MCM. (C) Two characteristics class-average, which show size variation. Class–averages were compared by subtraction (Small minus big) to verify that the eigenimage 7 was representative of the difference in size between small and big particles. Further MSA classification followed by multi–reference alignment (MRA) of the molecular images belonging to the both class–averages revealed a two ring-shaped class-averaged with a characteristic 8–fold and 7–fold symmetry for *Mth*MCM. Scale bar 100 Å.

Figure S3: 3D reconstruction strategy for the initial model of the full-length *Pab*MCM. **F**rom the top, 9 class averages were selected for the 3D reconstruction of an initial model of the full-length *Pab*MCM. The initial model was calculated by using the e2initial_model.py software from the EMAN2 image processing suite[21]. The best initial model, showed in figure, was chosen based by evaluating the correspondence between class averages (Av) and the re-projections (Re). The initial 3D model of the full-length *Pab*MCM single octamer was rendered in Chimera [38].

Figure S4: 2D image processing of cryo-EM images. A. Typical raw micrograph. B. Typical CTF image. D. individual particles. E. 2D classes from the full dataset. Individual images belonging to the classed in the red squares were selected and processed as a subdataset of single ring images. F. Classes calculated from the selected subset. Classes in red circles are consistent with those in Figure 2 and Figure S3. Boxsize=290 Å.

Supplementary Tables with legends:

Primer	Sequence 5' – 3'	Length
Oligo1	TATATACATATGGATAGAGAGGAGATCATCGAGAGATTCCTG	42
Oligo2	GCGCGGGGTACCTCAGACGGTTCTGTAATAACC	33
Oligo3	P–ACGGCCGCGGTGG	13
Oligo4	P–TTTCGCGACTCCCGG	15
Oligo5	GATCCGGGAGTCGCGAAAAGCCAACTTCTCAGATAC	36
Oligo6	CTAACCACCGCGGCCGTGAGCCCAGCGGCAGAACTC	36
Oligo7	6FAM-CAAGCAGTCCTAACTTTGAGGCAGAGTCCCCCACCTAACTTTAA	45
Oligo8	TTAAAGTTAGGTGGGGGACTCTGCCTCAAGACGGTAGTCAACGTGACCGCAGCAAACCTG	60

Table 1: List of oligonucleotides. Primers used to PCR-amplify the N-terminus of MCM (Oligo 1 and Oligo 4), the fragment Ser499-Leu525 (Oligo5 and Oligo6); and the C-terminal fragment of MCM (Oligo3 and Oligo2).

Oligo	Sequence 5' – 3'	Length
OligoA	TTTTTTTTTTTTTTTTTTTTTTTTTTTTTTTTTTTTTTTTTTTTGCTCGTGCAGACGTCG AGGTGAGGACGAGCTCCTCGTGACCACG	88
OligoB	Atto647-CGTGGTCACGAGGAGCTCGTCCTCACCTCGACGTCTGCACGAGCTTT TTTTTTTTTTTTTTTTTTTTTTTTTTTTTTTTTTTTTTTTTT	88
OligoC	CGTGGTCACGAGGAGCTCGTCCTCACCTCGACGTCTGCACGAGC-Trap*	44

Table 2: List of oligonucleotides. Oligonucleotides used for the helicase assay in Figure 1D.
